# Quantitative Diagnosis of Atrophic Gastritis by Probe-Based Confocal Laser Endomicroscopy

**DOI:** 10.1155/2020/9847591

**Published:** 2020-03-02

**Authors:** Xiaoyun Yu, Jie Chen, Liduan Zheng, Jun Song, Rong Lin, Xiaohua Hou

**Affiliations:** ^1^Department of Gastroenterology, Union Hospital, Tongji Medical College, Huazhong University of Science and Technology, Wuhan 430022, China; ^2^Department of Pathology, Union Hospital, Tongji Medical College, Huazhong University of Science and Technology, Wuhan 430022, China

## Abstract

**Aims:**

The aims of this study were to characterize nonatrophic and atrophic gastric mucosa under conventional endoscopy and probe-based confocal laser endomicroscopy (pCLE) modes and to define quantitative diagnostic parameters for these lesions under pCLE.

**Method:**

In phase I, 64 patients with gastric mucosal lesions diagnosed by gastrointestinal endoscopy were enrolled in the study. Normal mucosa and suspicious lesions were evaluated under normal white light imaging (WLI) and pCLE mode. Descriptive characteristic of gastric mucosal inflammation and atrophy under pCLE were defined according to the histology. In phase II, the criteria for nonatrophic gastritis (NAG) and chronic atrophic gastritis (CAG) under pCLE were used to diagnose the mucosal lesions in 431 patients. Diagnostic accuracy of each endoscopy modes was evaluated by measuring the concordance with histology.

**Result:**

A total of 64 patients with 187 positions were enrolled in the first part of this study. According to the histological diagnosis, the vessel diameter was increased in the NAG (11.18 ± 0.1 *μ*m) and CAG (13.21 ± 0.29 *μ*m) and CAG (13.21 ± 0.29 *μ*m) and CAG (13.21 ± 0.29 *μ*m) and CAG (13.21 ± 0.29 *μ*m) and CAG (13.21 ± 0.29 *μ*m) and CAG (13.21 ± 0.29 *μ*m) and CAG (13.21 ± 0.29

**Conclusion:**

pCLE shows high potential for the diagnosis of gastric inflammation and atrophy based on quantitative criteria and has the ability to be a substitute for histology in the diagnosis of diffuse lesions in the stomach.

## 1. Introduction

Atrophic gastritis (AG) is defined as the loss of appropriate glands with/without replacement by intestinal-type epithelium and fibrous tissue. The relationship between atrophic gastritis and gastric cancer has been established in numerous studies [1–3]. It is important to screen for gastric mucosa atrophy during endoscopy.

Conventional white light imaging (WLI) endoscopy cannot accurately differentiate and diagnose mucosal atrophy and intestinal metaplasia. Histology is the gold standard in diagnosing gastric lesions during traditional endoscopy. According to the updated Sydney system, at least five nontargeted biopsies (from the incisura and the lesser and greater curvature of both the gastric antrum and the corpus) and target biopsies of lesions should be taken for adequate assessment of gastric conditions [4]. Some researchers even recommend seven or more specimen biopsies [5]. However, biopsy can still not assess the mucosa condition in site and in real time, and the mucosal injury caused by forceps is, in addition, difficult to avoid. Techniques are therefore needed to better differentiate normal from abnormal mucosa and facilitate the use of targeted, rather than random, biopsy protocols.

Confocal laser endomicroscopy (CLE) is an imaging technique enabling real-time and in vivo microscopic evaluation of tissues at the cellular level during standard endoscopic procedure. This technology can be used through endoscope-based CLE system (eCLE; Pentax, Tokyo, Japan) or through probe-based confocal laser endomicroscopy (pCLE) system (Cellvizio, Mauna Kea Technologies, Paris, France). Numerous studies have shown the applications of eCLE and pCLE for accurate detection of gastrointestinal lesions [6–10]. Wang et al.'s [11] and Liu et al.'s [12] studies described the characteristics of atrophic gastritis as decreased gastric pits and markedly dilated opening under eCLE system. Whether the diagnostic criterion of atrophic gastritis under pCLE system was the same as in eCLE system was not clear according to their different imaging acquisition rate and field of view. Li et al.'s [13] study showed a high efficiency in diagnosis atrophic gastritis using the criterion above under pCLE system. However, other study showed that the agreement between different doctors for pCLE finding was poor [14]. Therefore, quantitative criteria under pCLE system may provide better consistency in diagnosis gastric lesions.

Hence, the aims of this study were to prospectively assess the feasibility and efficacy of pCLE by defining criteria of gastric atrophy related to glandular cells spacing and amount, as well as vascular diameters.

## 2. Methods

### 2.1. Process

This study contained two phases. Firstly, patients enrolled in phase I underwent WLI and pCLE endoscopy. Lesions of gastric mucosa were observed under WLI and pCLE, respectively. Biopsy for histological diagnosis was performed, and pCLE images were reserved for further analysis. The characters of each lesion under pCLE were analyzed and matched to histology diagnosis. The criteria of chronic gastritis (with or without atrophy) were established according to the characters under both histology and pCLE image. In phase II, patients were performed with both WLI and pCLE. The diagnosis of gastric lesions was defined under both pCLE criteria and histology. The consistency of these two diagnosis criteria was analyzed.

### 2.2. Phase I

#### 2.2.1. Patients

Patients with normal gastric mucosa, chronic gastritis (CG) presence, or absence of mucosal atrophy who were diagnosed by gastrointestinal endoscopy and histology between January 2012 and May 2013 were enrolled in the study. Patients with acute gastrointestinal bleeding, severe organ failure, pregnancy, breastfeeding, and allergy to fluorescein and those who were receiving anticoagulant and antiplatelet drugs within 7 days of the study were excluded. All the patients participating to the examination provided signed informed consent.

#### 2.2.2. Endoscopy

All patients were prepared for routine gastroendoscopy. To remove any excess mucus on the gastric mucosal surface, 20 ml of saline solution containing 2 ml simethicone emulsion (Berlin-Chemie AG) was taken orally 5 min before the examination. All procedures were performed using Olympus GIF-HQ260Z magnifying video endoscope. Image stabilization was achieved by using a plastic cap at the endoscope tip. After topical sedation, the endoscope was gently inserted into the stomach. At first, the mucosal condition was evaluated under WLI. Gastric mucosal lesions, such as erosion, elevated, hyperemia, granular mucosa, and gray intestinal-type epithelium, were targeted. The atrophic mucosal area is of a grossly pale-yellowish color with granular or hyperemic surface and transparent blood vessels, while normal mucosa is reddish-colored and smooth [15, 16].

#### 2.2.3. Confocal Laser Endomicroscopy

Microscopic architecture of all suspicious lesions was observed in vivo, using pCLE. During the endoscopic procedure, the confocal miniprobe was inserted in the accessory channel of a conventional endoscope, down to the stomach. After observation by WLI mode, 5 ml of 10% fluorescein sodium solution (Baiyunshan Mingxing Pharmaceutical Co. Ltd., Guangzhou, China) was administered intravenously as a contrast agent. Once inserted, the distal tip of the miniprobe was placed in gentle contact with the targeted lesion, and in vivo microscopic images of the mucosa were obtained. The depth of imaging of the pCLE confocal miniprobe used in the study (GastroFlex UHD, Mauna Kea Technologies, France) ranges from 55 to 65 *μ*m below the surface, and the rate of image acquisition is 12 frames/s with a scanning field of 30,000 pixels. All the images of the mucosa were saved into a computer for further image analysis.

#### 2.2.4. Histology

Targeted biopsies of each examined area were performed. Biopsy specimens were fixed in 4% methanal and sectioned into 4 *μ*m thick samples. Paraffine sections were stained with hematoxylin and eosin (HE). To prevent interobserver variability, histological sections were graded by a single pathologist, who was blinded to the clinical data and endoscopy results. The diagnostic criteria of CG were based on the Updated Sydney System [4].

#### 2.2.5. Evaluation of Confocal Images

The images were obtained and analyzed after endoscopic diagnosis. All the images of gastric lesions were carefully observed by the endoscopist who was previously trained on the interpretation of confocal endomicroscopic imaging for 6 months with more than 500 pCLE images in stomach mucosa. She has worked on pCLE in GI tract (including upper and lower GI tract) alone for 6 months before conducting this study.

The analysis of the pCLE image was done offline. The endoscopist performed EGD, pCLE, and biopsy in real time. All the images of EGD and pCLE in each lesion were collected and numbered for offline analysis. The offline interpretations were performed after image collection for all the lesions. When interpreted, the sequence of each lesion was random, and the endoscopist did not know the information of EGD and the pathology result corresponding to a certain pCLE image.

The microarchitecture of glandular epithelial cells, the absence of goblet cells, the diameters of capillaries, and the space between adjacent glands were observed for each lesion. The diameter of capillaries was defined as the average diameter of five consecutive capillaries observed on the lesion. The space between glands was defined as the average distance between two adjacent glands observed on five consecutive glands.

#### 2.2.6. Analysis of pCLE Images and Histology Diagnosis

All the biopsy tissues diagnosed as normal, NAG, and CAG based on the Updated Sydney System were focused on. The diameters of capillaries and space between glands from pCLE images corresponding to the diagnosis were calculated. The diagnostic criteria of CAG would be established and used for further diagnosis in phase II of this study.

### 2.3. Phase II

Patients with CG presence or absence of mucosal atrophy who were diagnosed by gastrointestinal endoscopy and histology between July 2013 and July 2017 were enrolled in the study. After WLI endoscopy examination, all suspicious lesions were observed in vivo using pCLE. According to the diagnosis criteria above, endoscopist would determine the diagnosis under pCLE in real time. Finally, targeted biopsies of these lesions were performed. The consistency of pCLE and histology diagnosis criteria was statistically analyzed. The endoscopists and pathologist were blind to the diagnosis from each other.

### 2.4. Statistical Analysis

The statistical analysis was performed by the statistical software package SPSS 19.0. The diameters of capillaries and the space between glands in different mucosa type were calculated and shown as mean ± SE. The efficacy of parameters under pCLE for the diagnosis of atrophy was evaluated by the area under the receiver operating characteristic (ROC) curve analysis in the first part of this study. In phase II, the McNemar test was used to examine the concordance of endoscopic diagnostic modes and histology. A *P* value of 0.05 was considered statistically significant. The agreement was regarded as poor with Kappa values below 0.4, good with values between 0.4 and 0.75, and excellent with values over 0.75.

## 3. Results

### 3.1. General Information

A total of 64 patients with 187 positions were recruited into phase I of this study, and 431 patients in phase II. Patients in phase I included 37 males and 27 females, with a mean age of 49.37 years (ranging from 17 to 70 years). 187 positions with 58 as normal mucosa, 64 as CG, and 65 as CAG diagnosed by WLI were observed under pCLE and were sampled for histological diagnosis. In phase II, there were 431 patients enrolled into the study with 232 males and 199 females. The mean age was 51.47 years (ranging from 22 to 78 years). A total of 431 positions were observed in this part.

### 3.2. Phase I

Among the 187 sites from 64 patients in this part, 50 were diagnosed as normal, 95 as CG, and 42 as CAG (13 as mild, 20 as moderate, and 9 as severe atrophy) by histology. The images of each position under pCLE were analyzed according to the histology diagnosis.

#### 3.2.1. Imaging Features of Gastric Mucosa under pCLE

**Figure 1:**
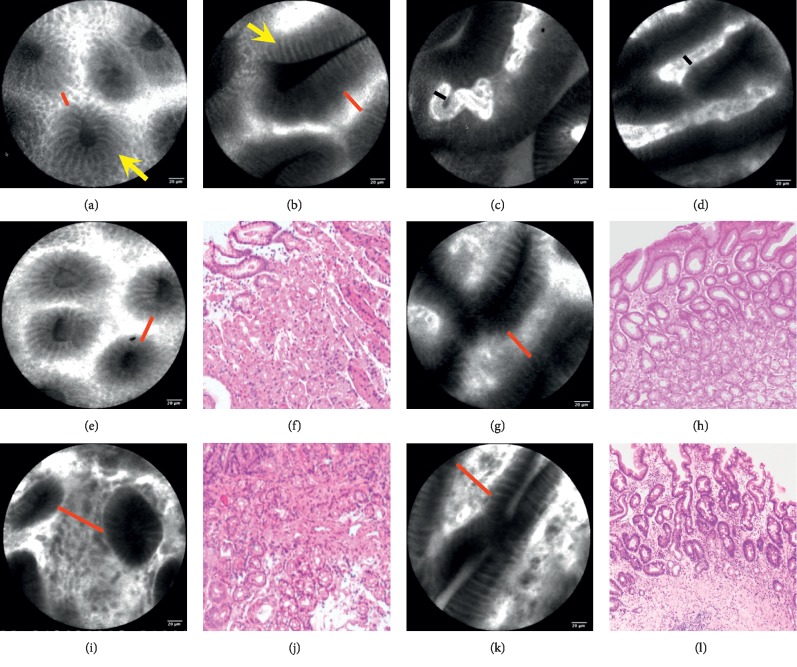
Imaging of gastric mucosa under histology and pCLE. The pCLE images of normal gastric mucosa showed round pit patterns as well as subepithelial capillaries surrounded by honeycomb-like structures in the corpus (Figure 1(a)) and rhabditiform pit patterns and coil-shaped network of capillaries in antrum (Figure 1(b)). The subepithelial capillaries were highlighted because of the flowing of fluorescein within the vessels (black line in Figure 1(c)). In inflammatory mucosa, the fluorescein sodium permeated into the interstitial space and showed the highlighted space among the glands (black line in Figure 1(d)). Among the lesions diagnosed as NAG ((f) in corpus and (h) in antrum) and CAG ((j) in corpus and (l) in antrum) by histology, the pCLE images showed increased distance between glands (interstitial space between two adjacent glands, red lines). (e) and (g) show increased distance between glands in corpus and antrum in NAG group. (i) and (k) show that in CAG group.

The pCLE images of normal gastric mucosa showed round pit patterns as well as subepithelial capillaries surrounded by honeycomb-like structures in the corpus (Figure 1(a)) and rhabditiform pit patterns and coil-shaped network of capillaries in antrum (Figure 1(b)). The subepithelial capillaries were highlighted because of the flowing of fluorescein within the vessels (black line in Figure 1(c)). Among the normal mucosa (*N* = 50), the vessel diameter was 10.58 ± 0.13 *μ*m and the distance between glands (Figure 1, red line) was 17.75 ± 0.51 *μ*m (see Table 1).

Among the lesions diagnosed as NAG and CAG, the mucosal images showed increased vessel diameter (Figure 1(d)) and distance between glands (Figure 1, red lines). In NAG group, the vessel diameter was 11.18 ± 0.1 *μ*m (*N* = 95, *P* > 0.05 vs. normal, and *P* < 0.05 vs. CAG group, Table 1), and the distance between glands was 22.38 ± 0.45 *μ*m (*P* < 0.05 vs. normal and CAG group). The parameters in CAG group were 13.21 ± 0.29 *μ*m (*N* = 42, *P* < 0.05 vs. normal group and NAG group) and 34.66 ± 0.82 *μ*m (*P* < 0.05 vs. normal group). The parameters among mild, moderate, and severe atrophy in CAG group had no significant difference (each *P* > 0.05).

#### 3.2.2. Diagnostic Criteria of Gastritis under pCLE

A specialty of pCLE system was its measuring scale (20 *μ*m) at the bottom right corner of the image. The endoscopists could evaluate some certain parameters under real time following this scale. The present study measured the vessel diameter and the distance between glands after endoscophic procedure by Digimizer image analysis software. According to the parameters above, the vessel diameter and the distance between glands had a significant difference among normal, CG, and CAG groups. However, neither the distance between glands in normal and CG group nor the vessel diameter among three groups was difficult to distinguish under real-time procedure because the difference was not too big. The only parameter which was easily distinguished was the distance between glands in CAG group compared to the other two groups. We selected the certain cutoff value for the discrimination of CAG and nonatrophic mucosa.

The best cutoff value between atrophic mucosa and nonatrophic mucosa was >27.5 *μ*m in distance between glands. The areas under the ROC curve (AUC) were 0.979, with a sensitivity and specificity of 95.2% and 92.4%. However, it was difficult to measure the distance of 27.5 *μ*m by the scale under the image. If the cutoff value between CAG and nonatrophic mucosa was >30 *μ*m in distance between glands, the sensitivity and specificity should be 85.7% and 93.8%.

According to the parameters above, we selected the certain cutoff value for the discrimination of nonatrophic gastritis and CAG. The optimal cutoff value between nonatrophic gastritis and CAG was >30 *μ*m in distance between glands.

### 3.3. Phase II

#### 3.3.1. Utilization of Diagnostic Criteria on Nonatrophic Gastritis and Atrophic Gastritis

Among the 431 positions observed in this part, 86 were diagnosed as NAG and 345 as CAG under WLI. However, 184 of them were diagnosed as NAG and 247 as CAG under histology. The sensitivity, specificity, PPV, and NPV of WLI were 95.6%, 41.0%, 68.7%, and 87.2% (see Table 2). Distinguishing atrophic mucosa from the nonatrophic one under WLI had a significant difference compared to histology (McNemar *P* < 0.05, Table 2). According to the diagnostic criteria in part 1, there were 169 positions diagnosed as nonatrophic gastritis and 262 as atrophic gastritis under pCLE. The sensitivity, specificity, PPV, and NPV were 90.3%, 78.8%, 85.1%, and 85.8%. Distinguishing the atrophic mucosa from nonatrophic one under pCLE had no significant difference compared to histology (McNemar *P*=0.077). The consistency of pCLE (Kappa value = 0.698) with histology was much better than WLI (Kappa value = 0.393).

## 4. Discussion

In this study, we analyzed the mucosal characteristics of atrophic gastritis under WLI and pCLE modes. Using quantitative criteria for pCLE such as the distance between glands allowed evaluating the type of chronic gastritis in vivo and in real time.

Conventional endoscopy is not frequently correlated with histological alterations for predicting histological gastritis [17]. The concordance of CAG diagnosed by WLI was poor compared to histology in the present study. WLI could only show the granular mucosa and visualization of submucosal blood vessel, with or without gray intestinal-type epithelium, which refers to severe atrophy or intestinal metaplasia, but had no advantage on nonobvious lesions. WLI without biopsy is incomplete in terms of diagnosis of gastric mucosa atrophy. Combination of the NBI system and magnifying endoscopy allows for clear visualization of microscopic structures of the mucosal pit patterns and capillary patterns [18]. Ridged surface structures encasing the dilated coiled subepithelial capillaries (SECs) and villous to granular surface structures under NBI promoted mucosal atrophy and intestinal metaplasia [19]. However, the tubulovillous pits with clearly visible coiled or wavy vessels could only predict the occurrence of atrophic gastritis with 50.0% sensitivity and 96.3% specificity in Tahara et al.'s study [20]. Similarly, the efficiency of NBI or chromoendoscopy to diagnose atrophic gastritis was about 70%, compared to histology in Liu et al.'s study [12]. This may be caused by their limits as these techniques cannot show a microscopic characterization of the mucosa and do not provide information on the cellular structures of the observed tissue.

The present CLE which permits microscopic visualization of the mucosa during endoscopy at an approximately 1000-fold magnification provides a new method to diagnose gastric lesions directly. A number of studies have investigated the characteristics of mucosal pits and vessels in gastritis and precancerous and gastric cancer in the past years [6–10, 21]. A recent study described the imaging of CAG as a decreased number of pits and dilation of pits prominently using endoscope-based CLE (eCLE) [11]. However, the field of view under pCLE system was smaller than that under eCLE. In Wang's study, the mean number of gastric pits in one image was about 20 in normal mucosa and decreased to 5 in CAG mucosa. These data in our study was about 5 in normal mucosa and 2 to 3 in CAG mucosa (data not shown). Therefore, the diagnostic criteria of CAG under eCLE and pCLE will not be the same.

A specialty of pCLE system compared to eCLE was its measuring scale (20 *μ*m) at the bottom right corner of the image. The endoscopists could evaluate some certain parameters under real time following this scale. With higher imaging acquisition rate and 1000-fold magnification in a smaller field of view, the outline of capillary and the space between glands were clear under pCLE system. Combined with its measuring scale, the endoscopists could easily measure the capillary diameter and the distance between glands in different lesions.

In this study, we measured the capillary diameter and the distance between glands to evaluate the imaging feature under pCLE and matched these parameters to histological diagnosis. The capillary diameter was 8–10 *μ*m in normal mucosa under pCLE mode and increased significantly in chronic gastritis with/without atrophy. The infiltration of inflammatory cells was also conspicuous, and the distance between consecutive glands widened in atrophic mucosa.

Although the vessel diameter and the distance between glands had a significant difference among normal NAG and CAG groups (*P* < 0.05), neither the distance between glands in normal and NAG group (17.75 ± 0.51 *μ*m vs. 22.38 ± 0.45 *μ*m) nor the vessel diameter among the three groups (10.58 ± 0.13 *μ*m, 11.18 ± 0.1 *μ*m, and 13.21 ± 0.29 *μ*m in normal, NAG, and CAG group) was difficult to distinguish under real-time procedure because the difference was not too big. So, we chose the distance between glands as the parameter to distinguish atrophic mucosa from nonatrophic mucosa. When the distance between glands was >30 *μ*m, the sensitivity for the diagnosis of atrophy will be 90.3% and had good agreement with histology (Kappa value = 0.698).

This method was also easy for used. The measuring scale at the bottom right corner of the image showed a 20 *μ*m length. It was easy to estimate whether the distance between glands was more than 30 *μ*m among different endoscopists. However, this method could not tell the difference among mild, moderate, and severe atrophic gastritis.

In conclusion, the present study compared the diagnostic efficacy of WLI and pCLE in the classification of gastritis. The new pCLE technique showed significant superiority to visualize microvascular structure, gland pattern, epithelial shapes, and goblet cells. The establishment of quantitative diagnostic criteria on atrophic gastritis will ease the evaluation of lesions in real time by endoscopists and will allow the decrease of injuries caused by biopsy forceps. Although pCLE itself has still some limitations, such as the limited observed depth and the unclear display of nucleus, it is a promising procedure for accurate histological evaluation in vivo, especially in the diagnosis of gastric diffuse lesions, where it is capable to be a substitute for biopsy.

## Figures and Tables

**Table 1:** Imaging parameters of gastric mucosa under pCLE.

		Vessel diameter (*μ*m)	Distance between glands (*μ*m)
Normal	*n* = 50	10.58 ± 0.13	17.75 ± 0.51
NAG	*n* = 95	11.18 ± 0.1	22.38 ± 0.45^*∗*^
CAG	*n* = 42	13.21 ± 0.29^*∗*#^	34.66 ± 0.82^*∗*#^
	Mild (*n* = 13)	13.25 ± 0.46	32.5 ± 1.13
	Moderate (*n* = 20)	13.44 ± 0.46	35.27 ± 1.25
	Severe (*n* = 9)	12.67 ± 0.67	36.431.91

^#^ *P* < 0.05 vs. NAG group. ^*∗*^*P* < 0.05 vs. normal group.

**Table 2:** Diagnostic efficiency of pCLE and WLI on atrophic or nonatrophic gastritis.

		Histology (*n*)				Sensitivity (%)	Specificity (%)	PPV (%)	NPV (%)
		NAG	CAG	Kappa	*P* (McNemar)				
pCLE (*n*)	NAG	145	24			90.3	78.8	85.1	85.8
	CAG	39	223	0.698	0.07			

WLI (*n*)	NAG	75	11		<0.05	95.6	41.0	68.7	87.2
	CAG	108	237	0.393				

## Data Availability

The data used to support the findings of this study are included within the article.
